# In Situ/Microinvasive Adenocarcinoma of the Uterine Cervix and HPV-Type Impact: Pathologic Features, Treatment Options, and Follow-Up Outcomes—Cervical Adenocarcinoma Study Group (CAS-Group)

**DOI:** 10.3390/cancers15112876

**Published:** 2023-05-23

**Authors:** Luca Giannella, Giovanni Delli Carpini, Jacopo Di Giuseppe, Giorgio Bogani, Francesco Sopracordevole, Nicolò Clemente, Giorgio Giorda, Rosa Pasqualina De Vincenzo, Maria Teresa Evangelista, Barbara Gardella, Mattia Dominoni, Ermelinda Monti, Chiara Alessi, Lara Alessandrini, Alessio Pagan, Marta Caretto, Alessandro Ghelardi, Andrea Amadori, Massimo Origoni, Maggiorino Barbero, Francesco Raspagliesi, Tommaso Simoncini, Paolo Vercellini, Giovanni Scambia, Andrea Ciavattini

**Affiliations:** 1 Woman’s Health Sciences Department, Gynecologic Section, Polytechnic University of Marche, 60123 Ancona, Italy; lucazeta1976@libero.it (L.G.); giovdellicarpini@gmail.com (G.D.C.); jacopodigiuseppe@gmail.com (J.D.G.); 2 Gynecological Oncology Unit, Fondazione IRCCS—Istituto Nazionale Tumori, 20133 Milan, Italy; giorgio.bogani@istitutotumori.mi.it (G.B.); raspagliesi@istitutotumori.mi.it (F.R.); 3 Gynecologic Oncology Unit, IRCCS—Centro di Riferimento Oncologico di Aviano, 33081 Aviano, Italy; fsopracordevole@cro.it (F.S.); nicoclemente@alice.it (N.C.); ggiorda@cro.it (G.G.); 4 Dipartimento Scienze della Salute della Donna, del Bambino e di Sanità Pubblica, UOC Ginecologia Oncologica, Fondazione Policlinico Universitario A. Gemelli IRCCS, 00168 Rome, Italy; rosa.devincenzo@unicatt.it (R.P.D.V.); mariateresa.evangelista@policlinicogemelli.it (M.T.E.); giovanni.scambia@policlinicogemelli.it (G.S.); 5 Dipartimento di Scienze della Vita e Sanita Pubblica, Università Cattolica Del Sacro Cuore, 00168 Rome, Italy; 6 Department of Obstetrics and Gynecology, Fondazione IRCCS Policlinico San Matteo, Università degli Studi di Pavia, 27100 Pavia, Italy; barbara.gardella@gmail.com (B.G.); matti.domino@gmail.com (M.D.); 7 Department of Clinical Sciences and Community Health, Fondazione IRCCS Ca’ Granda, Ospedale Maggiore Policlinico, 20122 Milan, Italy; ermelindamonti@gmail.com (E.M.); paolo.vercellini@unimi.it (P.V.); 8 UOC Ostetricia Ginecologia, Dipartimento per la Salute della Donna e del Bambino, Azienda Ospedaliera—Università di Padova, 35128 Padova, Italy; chiara.alessi@aopd.veneto.it; 9 Pathological Anatomy Unit, Department of Medicine DIMED, University of Padova, 35128 Padova, Italy; lara.alessandrini@aopd.veneto.it; 10 ULSS 2 Marca Trevigiana, 31100 Treviso, Italy; alessio.pagan@aulss2.veneto.it; 11 Division of Obstetrics and Gynecology, Department of Clinical and Experimental Medicine, University of Pisa, 56126 Pisa, Italy; martacaretto@gmail.com (M.C.); tommaso.simoncini@med.unipi.it (T.S.); 12 Azienda Usl Toscana Nord-Ovest, UOC Ostetricia e Ginecologia, Ospedale Apuane, 54100 Massa, Italy; ghelardi.alessandro@gmail.com; 13 Gynecology Unit, Ospedale di Forlì, 47121 Forlì, Italy; dott.amadori@gmail.com; 14 Department of Gynecology & Obstetrics, Vita Salute San Raffaele University School of Medicine, 20132 Milan, Italy; origoni.massimo@hsr.it; 15 Department of Obstetrics and Gynecology, Asti Community Hospital, 14100 Asti, Italy; barberom@tin.it

**Keywords:** in situ adenocarcinoma, microinvasive adenocarcinoma, cervix, human papillomavirus, recurrence, follow-up, treatment

## Abstract

**Simple Summary:**

The impact of human papillomavirus (HPV) status on cervical glandular lesions is a debated topic. In general, non-HPV-related adenocarcinomas would appear to have a worse prognosis. Assessing this question in early stage or in situ adenocarcinomas may be interesting, as conservative surgery is feasible in these cases. Moreover, this population group accounts for 80% of the cases of high-grade glandular lesions in clinical practice. This research aims to evaluate the outcomes of long-term follow-up in HPV-positive and -negative women. Evaluating these findings may be of interest to know whether HPV status may impact management planning in the early and in situ stages of adenocarcinomas. Our results showed that the recurrence rate was not significantly different between the two groups. However, an analysis limited to only a portion of our sample showed a type-specific association with disease relapse.

**Abstract:**

It is unknown whether human papillomavirus (HPV) status impacts the prognosis of early stage cervical glandular lesions. This study assessed the recurrence and survival rates of in situ/microinvasive adenocarcinomas (AC) according to HPV status during a 5-year follow-up. The data were retrospectively analyzed in women with available HPV testing before treatment. One hundred and forty-eight consecutive women were analyzed. The number of HPV-negative cases was 24 (16.2%). The survival rate was 100% in all participants. The recurrence rate was 7.4% (11 cases, including four invasive lesions (2.7%)). Cox proportional hazards regression showed no difference in recurrence rate between HPV-positive and HPV-negative cases (*p* = 0.148). HPV genotyping, available for 76 women and including 9/11 recurrences, showed a higher relapse rate for HPV-18 than HPV-45 and HPV-16 (28.5%, 16.6%, and 9.52%, *p* = 0.046). In addition, 60% and 75% of in situ and invasive recurrences, respectively, were HPV-18 related. The present study showed that most ACs were positive for high-risk HPV, and the recurrence rate was unaffected by HPV status. More extensive studies could help evaluate whether HPV genotyping may be considered for recurrence risk stratification in HPV-positive cases.

## 1. Introduction

The rates of cervical cancer in industrialized countries have decreased in the last twenty years, though it represents one of the leading causes of cancer death in developing countries [1]. This sharp decline is due to primary and secondary prevention that has been added with the introduction of HPV vaccines over the past 20 years [2,3]. Against this general reduction in cervical cancer, the rates of pre-invasive and invasive glandular lesions are increasing [4]. Conversely, for squamous lesions, there was a reduction in invasive cancers but an increase in in situ lesions [4]. These differences between glandular and squamous lesions may be due to a delayed diagnosis of cervical glandular lesions, to a faster transition from pre-invasive forms, such as adenocarcinoma in situ (AIS), to invasive ones, or to pathogenetic processes different from squamous lesions [5,6,7].

Another difference between cervical adenocarcinoma (CA) and squamous cell cancer is the relationship with HPV infection. While squamous lesions are predominantly related to HPV infection, AC has a frequency of HPV-negative lesions ranging from 10 to 20% [4,8]. Glandular lesions are mainly related to HPV-18 [9]. More specifically, it is present in 38–50% of AIS cases and up to 50% in invasive lesions [9]. In this regard, in 2018, the International Endocervical Criteria and Classification (IECC) divided cervical glandular pathologies into HPV-related and non-HPV-related lesions [4]. Several studies have reported HPV-negative glandular lesions to have a worse prognosis than their HPV-related counterpart lesions [4,10,11].

In a recent study including 341 surgical specimens of AC, 100% of non-HPV-related lesions (negative for high-risk HPV including HPV16, 18, 26, 31, 33, 35, 39, 45, 51, 52, 53, 56, 58, 59, 66, 68, 73 and 82) were classified as Silva Pattern C (the worst prognostic pattern) [10]. Conversely, a study including 113 women with AC (stages I–IV) found no impact of HPV during follow-up on survival rate. In that report, women were categorized based on high-risk vs. non-high-risk HPV positivity. Only HPV-type-45 showed a shorter 5-year survival than HPV-16 or -18 [12]. Based on these data, the topic of HPV status and follow-up outcomes seems controversial.

Given that for in situ/microinvasive glandular lesions, conservative surgery is feasible as an alternative to standard treatment, it may be of interest also to assess the HPV status impact in these early stages. The evaluation of long follow-up outcomes in in situ and microinvasive stages of the cervical glandular lesions according to HPV status (positive vs. negative) is lacking.

The present study aimed to assess the recurrence and survival rates in women with in situ/microinvasive AC according to HPV status. Secondly, it assessed the type-specific HPV recurrence and survival rates.

## 2. Materials and Methods

This retrospective multi-institutional study included women with a histological diagnosis of AIS or micro-invasive AC (stage 1A) on cone or hysterectomy specimens. All participants were treated at 13 oncology referral Centers between January 2012 and December 2016 with a complete follow-up of 5 years. All women had a screening HPV testing dated no more than two months before conization. Further definitive treatments were to be at most two months after the first conization. All patients with previous conizations, immunological disease, and unavailable HPV testing before surgery were excluded. The participating departments are research centers managing women included in both opportunistic and organized cervical cancer screening programs.

Based on the Italian law on non-interventional observational studies, the Ethics Committee (Comitato Etico Regionale Marche) took note of the study (Prot. 2022/146) [13]. Furthermore, based on the provisions of the Italian law on non-interventional observational studies, the consent of the patients is not an essential condition [13]. The present study was registered at Clinical-Trials.gov—Identifier: NCT05267834.

Based on pre-treatment HPV testing results via Hybrid Capture 2 (including genotypes 16/18/31/33/35/39/45/51/52/56/58/59/68), women were divided into HPV-positive vs. HPV-negative. 

The following variables were collected: age, menopausal status, parity, smoking habit, HPV vaccination status, conization type (cold knife conization, loop electrosurgical excision procedure, laser conization), cone length (mm), pre-treatment cytology results, definitive treatment (fertility-sparing vs. standard treatment), adnexa treatment, lymphovascular space status, stage (1A1, 1A2, AIS), histology (usual type, Mucinous-NOS, gastric type, intestinal type, signet ring cell, villoglandular, endometrioid, clear cell, serous, and mesonephric), HPV status and cytology during follow-up, recurrence rate (categorized as CIN2/3, AIS, VAIN2/3, invasive cancer), survival rate, and time to recurrence (months). 

We included pre-treatment HPV genotyping in the analysis performed in eight of the thirteen centers participating in the study. Since most cervical glandular lesions are due to HPV-16/18/45, the genotypes have been divided into HPV 16, HPV-18, HPV-45, other HPVs, and multiple HPV infections [14]. 

According to the study period, the histopathological diagnosis of stage 1A refers to the 2014 FIGO staging [15]. Similarly, histopathology refers to the 2014 WHO classification [16].

All data were retrieved from the electronic database used in our clinics and anonymized before analysis. HPV genotyping was performed using the HPV Sign^®^ Genotyping Test (Qiagen, Hilden, Germany), INNO-LiPA^®^ HPV Genotyping Extra assay (Innogenetics, Ghent, Belgium), or CLART^®^ HPV2 PCR (Genomica, Madrid, Spain).

Fertility-sparing treatment for women with stage AIS or 1A1 without LVSI included conization with negative margins; stage 1A1 with LVSI or 1A2 included conization with negative margins + pelvic lymph node dissection [17]. Standard treatment was administered for women with stage AIS or 1A1 without LVSI included extrafascial hysterectomy; for stage 1A1 with LVSI or 1A2, treatment included modified radical hysterectomy + pelvic lymph node dissection [17]

Follow-up was standardized for all women: co-testing + colposcopy every six months for three years, then co-testing + colposcopy annually for two years [18]. 

Categorical variables were expressed as numbers and percentages. The chi-squared test was used to compare categorical variables. Continuous variables were tested for normal distribution using the Kolmogorov–Smirnov test. The variables were expressed as median and interquartile range or media and standard deviation according to distribution. As appropriate, continuous variables were assessed using Student’s *t*-test or Mann–Whitney test. The univariate analysis compared independent variables according to HPV status. Based on the results of univariate analysis, follow-up outcomes based on HPV status were measured using Cox proportional hazards regression. The Cox proportional hazards regression model included significant explanatory variables in univariate analysis, in addition to HPV status and treatment type. The Kaplan–Meier survival analysis with the Log-rank test assessed the recurrence rate according to HPV genotypes.

Given that we included women with no follow-up losses, sample size calculation was performed using the estimation of a confidence interval with a required width for a single proportion based on the primary outcome: recurrence rate in in situ/microinvasive ACs. The literature reports a range of disease relapses between 2 and 14% [19,20,21,22,23]. We expected a mean value of 8%. With a confidence level of 95% and confidence interval width (2-sided) equal to 10 (±5%), the minimum required sample size should include 118 women.

MedCalc Statistical Software was used to perform statistical analyses (MedCalc^®^ Statistical Software version 20.218 (MedCalc Software Ltd., Ostend, Belgium; https://www.medcalc.org, accessed 1 April 2023). A value of *p* < 0.05 was considered statistically significant.

## 3. Results

**Figure 1:**
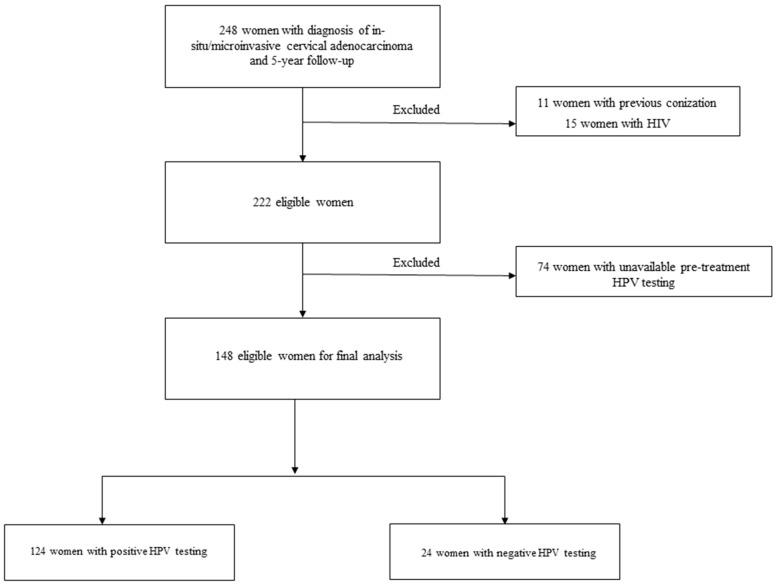
Study flow-chart.

Two hundred forty-eight consecutive women with in situ/microinvasive and a 5-year follow-up was included during the study period. After excluding 100 cases, 148 eligible women were analyzed (Figure 1). In total, of 11 women were excluded for previous conizations, 15 were excluded because they were immunocompromised, and, finally, 74 patients were excluded due to the unavailability of pre-treatment HPV testing.

Patient characteristics are reported in Table 1. The number of HPV-positive women was 124 (83.8%), whereas that of HPV-negative patients was 24 (16.2%). In total, 17.6% of women were in menopause, while 44.6% of patients were nulligravid; most women (68.2%) underwent loop electrosurgical excision procedures as initial conization type. The median cone length was 16.5 mm. Most women (62.2%) did not perform salpingo-oophorectomy. Lymphovascular space involvement was present in 16 women (10.8). A total of 85 (57.4%) women and 63 (42.6%) were administered fertility-sparing or standard treatment, respectively. Disease stage was 1A1, 1A2, AIS in 23 (15.5%), 21 (14.2%), and 104 (70.3%) women, respectively. Ten women (6.8%) were vaccinated using opportunistic anti-HPV vaccination before the development of cervical pathology. Most cases showed usual-type histopathology (120 women, 81.1%). The recurrence rate was 7.4% (11 cases, including four invasive lesions (2.7%)). Positive hr-HPV in follow-up showed a rate of 19.6%. The mean time to recurrence was 33.0 ± 22.9 months. The mean time to HPV positivity during follow-up was 26.4 ± 18.9. The survival rate was 100% in all cases.

The univariate analysis results comparing HPV-positive vs. HPV-negative women are reported in Table 2. Significant differences were found for age, menopausal status, LVSI, histopathology, and positive HPV testing in follow-up (Table 2). The median age in HPV-positive vs. HPV-negative women was 39.5 vs. 47, respectively (*p* = 0.032). Menopausal status was present in 14.5% vs. 33.5% of HPV-positive vs. HPV-negative women, respectively (*p* = 0.027). HPV-positive women had a rate of LVSI of 7.3% vs. 29.2% of HPV-negative women, respectively (*p* = 0.001). Histopathology showed a “usual type” histology in 83.9% of HPV-positive women vs. 66.7% of HPV-negative patients, respectively (*p* = 0.049). Finally, HPV positivity during follow-up was 22.6% in HPV-positive women vs. 4.2% in HPV-negative women, respectively (*p* = 0.038). No significant differences were found in parity, smoking habit, HPV vaccination status, conization type, cone length, pre-treatment cytology, definitive treatment, salpingectomy or salpingo-oophorectomy, stage, positive pap test in follow-up, recurrence rate including pre-invasive and invasive disease, and time to recurrence. HPV positivity during follow-up occurred at 19.5 ± 16.2 vs. 29.1 ± 19.5 months in women with and without recurrence (*p* = 0.22), respectively.

According to the results of univariate analysis and criteria reported in the method section, the Cox proportional hazards regression analysis is reported in Table 3, including the predictor variables (age, histopathology, menopausal status, lymphovascular space involvement, HPV test in follow-up, treatment type, and pre-treatment HPV status), coefficient b, standard error, Exp(b), and 95% confidence interval of Exp(b) (Table 3). Only positive HPV testing during follow-up showed an association with recurrence (*p* = 0.002).

**Figure 2:**
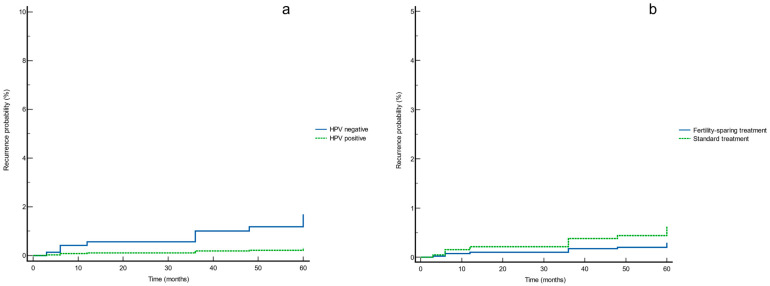
Survival curves for (**a**) HPV status and (**b**) treatment type according to the Cox proportional hazards regression analysis.

Based on the Cox proportional hazards regression results, the survival curves for HPV status and treatment type are reported in Figure 2. These showed no significant differences in recurrence rate ((a) *p* = 0.148 and (b) *p* = 0.294, respectively) (Figure 2).

HPV genotyping was available for 76 of 124 HPV-positive cases, including all recurrences in HPV-positive women (9 cases). Pre-invasive and invasive recurrences were related to HPV-18 in 3/5 cases (60%) and 3/4 cases (75%), respectively (Table 4).

**Figure 3:**
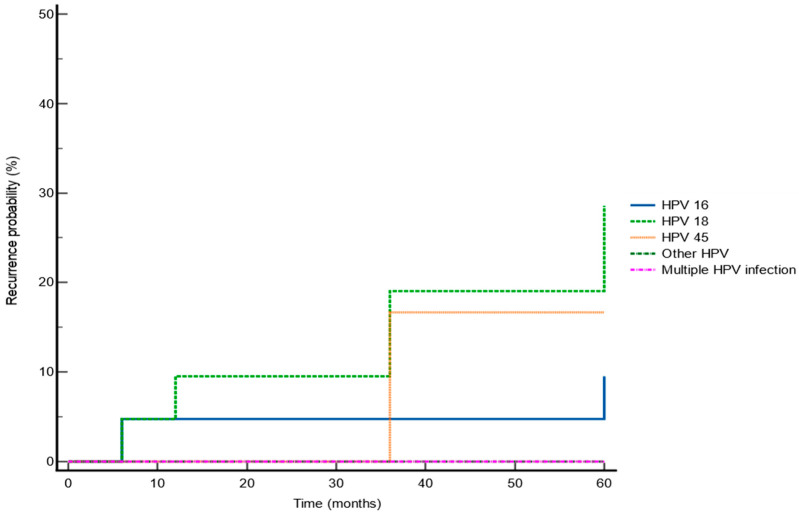
Kaplan–Meier survival analysis showing recurrence rate according to specific HPV-type.

The Kaplan–Meier survival analysis showed a higher recurrence rate for HPV-18 than HPV-16 and HPV-45 (28.57%, 9.52%, and 16.67%, respectively, *p* = 0.046) (Figure 3).

## 4. Discussion

The main findings of the present study showed that most ACs were HPV related, and the recurrence rate in in situ/microinvasive AC was unaffected by HPV status. Secondly, and limited to a fraction of our sample, HPV genotyping found that most preinvasive and invasive recurrences were HPV-18 related.

Cervical glandular lesions, unlike their squamous counterparts, are characterized by a higher rate of skip-lesions, and more aggressive behavior, resulting in a higher recurrence rate [24]. A wide range is described in the literature for relapses in in situ or microinvasive AC ranging from 2 to 14% [19,20,21,22,23]. The present study found a recurrence rate of 7.4%, including 2.7% of invasive relapse, within the range reported by previous studies. These numbers raise concerns about the possibility of conservative rather than standard treatment in women wishing to become pregnant. However, recent evidence has shown similar outcomes regarding recurrence and survival in women treated conservatively vs. standard options. In a study including 1567 women with microinvasive AC, radical treatment did not show better survival than conservative treatment [20]. Likewise, Liu et al. showed the efficacy and safety of cervical conization as a treatment option in 310 women with in situ AC [24]. In line with these results, our study also showed that the type of treatment did not affect the recurrence outcomes in HPV-positive and HPV-negative women. Concerning these data, however, it should be emphasized that our sample was primarily divided based on HPV status and not on follow-up outcomes.

Time to recurrence is another critical aspect of cervical glandular lesions. HPV clearance in adenocarcinoma has been reported to take longer than in squamous lesions; therefore, more extended surveillance is recommended [9,18]. Our results confirmed these data with an average time to recurrence of approximately three years. Furthermore, HPV test positivity was associated with disease relapse during follow-up in our study. These results confirmed those of previous authors who reported follow-up HPV status as the strongest predictor for recurrence in glandular lesions [9,18]. Conversely, positive cytology during follow-up did not correlate with recurrences. This finding underlines that HPV testing alone rather than co-testing may be the most cost-effective strategy to monitor these women after treatment. Additionally, for this result, it must be specified that the predictive variables inserted in the Cox proportional hazards regression model derived from the division of the sample based on the pre-treatment HPV status and not on the recurrence outcomes. Concerning HPV positivity during follow-up, it occurred on average earlier in women with recurrence, although there is no statistically significant difference. Likely, an earlier positive HPV test at follow-up may better reflect the “persistence” of the same genotype, which we know to be an independent risk factor for recurrences [25]. Conversely, a later positivity could be linked to a new acquisition of another HPV infection.

A further distinctive aspect of ACs is the link with HPV-negative cases [26]. Consistent with previous findings, our study’s rate of HPV-negative adenocarcinomas was 16%. In general, it is recognized that HPV-negative cervical lesions have worse outcomes than HPV-positive ones. However, in the literature, there are conflicting results. In a study including 136 women with cervical cancer, 10% were HPV-negative. The latter cases were more frequently adenocarcinomas with worse disease-free survival [27]. Conversely, a study including 51 patients with AC (stages I–IV) undergoing surgery with or without adjuvant or neoadjuvant therapy showed that HPV status did not impact survival rate. They showed HPV-16, -18, and -45 in 13, 18, and 3 of 43 HPV-positive tumors. In that study, only clinical stage and architectural grade were predictors of survival in AC [28]. Finally, Baalbergen et al. recently studied 113 women with AC (stages I–IV), including 86% at stage 1. They reviewed histological data and reassessed HPV status via PCR (including HPV16/18/31/33/35/39/45/51/52/56/58/59/66/68). The rate of HPV-positive AC in the early stages was 88%. The authors showed that recurrence and survival rate was unaffected by HPV status, except for HPV-45, which had worse outcomes than HPV-16 and -18. In that study, the HPV-18, -16, and -45 cases were 55, 37, and 7 out of 101 HPV-positives [12]. These results underline the utility of HPV testing in the detection of ACs. These data are relevant given that, currently, the screening test for the prevention of cervical cancer is the HPV test. Furthermore, survival outcomes would not appear to be influenced by the HPV genotype since HPV-45 cases were too few to be conclusive. However, the same authors suggest that their findings should be confirmed using more extensive studies [12].

There is scant literature on the impact of HPV status during long-term follow-up in in situ or microinvasive ACs. This population group is interesting as they account for more than 80% of high-grade cervical glandular lesions in clinical practice. Furthermore, they can be managed both conservatively and with standard treatment. Although previous studies have demonstrated comparable follow-up outcomes in women treated conservatively versus standard treatment in early stages of ACs, it is unknown whether this is also true for HPV-positive or -negative women with AIS or microinvasive adenocarcinoma.

Our results indicate that HPV status should not impact management planning, as there is no difference in follow-up outcomes. Although HPV-negative cases appear to have a higher recurrence rate after treatment, these results did not reach statistical significance (Figure 2, Table 3). Furthermore, in line with previous data, approximately 84% of ACs were hr-HPV related. These results underline the importance of HPV screening testing and HPV vaccination in cervical glandular lesions. Concerning the primary prevention of cervical cancer, there is a need to implement the coverage of HPV vaccination, given that most ACs are hr-HPV-positive and related to HPV genotypes included in HPV vaccines (HPV-16, HPV-18, and HPV-45) [29].

HPV genotyping included 9/11 recurrences and showed that HPV-18 was present in most preinvasive and invasive relapses. Previous studies have found HPV-18 as an adverse prognostic factor in cervical cancer. In 1067 early stage cervical cancers, HPV-18 positivity was an independent prognostic factor for disease relapse [30]. Furthermore, HPV-18 predicted worse outcomes in women with cervical cancer treated primarily with radiotherapy [31]. It is worth underlining that those previous studies did not differentiate cervical squamous from glandular lesions. More recently, in a study including 84 women with AIS, Belkic et al. showed HPV-18 positivity during follow-up as the best predictor for recurrence with an odds ratio of 141 [21]. In line with these data, the present study showed the type-specific impact of pre-treatment HPV in in situ and microinvasive ACs. These results must be interpreted cautiously since they were available only for just over 50% of cases. Furthermore, they included data from some and not all of the centers participating in the study. Therefore, their value remains limited. Further and more extensive studies using HPV genotyping should be performed to assess whether HPV-18 cases require more intensive and prolonged follow-up.

Interestingly, our study found only three cases of histopathologically non-HPV-related ACs. Consequently, the data on this type of ACs can be considered inconclusive. Furthermore, this underlines the rarity of these cases. On the other hand, this means that some of our HPV-negative cases had HPV-related histopathology. The explanation for this event may be due to false negative cases, or, as previously reported, they may be true negative HPV usual-type adenocarcinomas where HPV drives the initial transformation process; subsequently, it is lost in more advanced stages of transformation, and progressive somatic mutations play a crucial role [32,33].

The limitation of our study lies in its retrospective design. Additionally, there was no review of histopathology and HPV data using more sensitive methods. Genotyping was available for some women; therefore, these data should be taken cautiously. On the other hand, we have collected a fair number of women with rare conditions. The homogeneity of the sample, including well-standardized treatment options and long-term follow-up planning, should also be highlighted. Finally, being a multi-institutional study including numerous referral centers, the results are more generalizable.

## 5. Conclusions

The recurrence rate in in situ/microinvasive ACs was unaffected by HPV status. Therefore, decision making regarding the management of these women should not be based on this finding. During follow-up, HPV-18 was a recurrent factor for relapse. Given that the latter result is based on only a portion of the sample, more extensive studies could help evaluate whether HPV genotyping may be considered in HPV-positive cases for recurrence risk stratification in this population group.

## Figures and Tables

**Table 1:** Patient characteristics.

Independent Variables	n (%) (Sample Size = 148)
Age (years) (median and interquartile ranges)	40.5 (34.0–49.0)
Menopause	26 (17.6)
Nulligravid	66 (44.6)
Smoking habit	38 (25.6)
Vaccinated	10 (6.8)
Conization Type	
CKC	11 (7.4)
Laser conization	36 (24.3)
LEEP	101 (68.2)
Cone length (mm) (median and interquartile ranges)	16.5 (13.0–21.0)
Pap Test	
Negative	5 (3.4)
ASCUS/LSIL	18 (12.2)
ASCH+	94 (63.5)
AGC-NOS	19 (12.8)
AGC-FN	4 (2.7)
AIS	8 (5.4)
Definitive Treatment	
Fertility-sparing	85 (57.4)
Standard treatment	63 (42.6)
HPV status	
Positive	124 (83.8)
Negative	24 (16.2)
Adnexa	
Salpingo-oophorectomy	41 (27.7)
Salpingectomy	15 (10.1)
Not performed	92 (62.2)
Positive LVS	16 (10.8)
Stage	
1A1	23 (15.5)
1A2	21 (14.2)
AIS	104 (70.3)
Histopatology	
Clear cell	1 (0.7)
Endometrioid	1 (0.7)
Gastric type	1 (0.7)
Serous	1 (0.7)
Signet ring cell	1 (0.7)
Intestinal type	10 (6.8)
Mucinous-NOS	5 (3.4)
Usual type	120 (81.1)
Villoglandular	8 (5.4)
Positive hr-HPV in follow-up	29 (19.6)
Positive Pap test in follow-up	
ASCUS/LSIL	5 (3.4)
ASCH+	7 (4.7)
Recurrence	
AIS	4 (2.7)
VAIN 2/3	2 (1.3)
CIN 3	1 (0.7)
Invasive glandular	3 (2.0)
Invasive squamous	1 (0.7)
Time to recurrence (mean ± standard deviation, months)	33.0 ± 22.9
Time to HPV positivity (mean ± standard deviation, months)	26.4 ± 18.9

CKC: cold knife conization; LEEP: loop electrosurgical procedure; ASCUS: atypical squamous cells of undetermined significance; LSIL: low-grade squamous intraepithelial lesion; ASCH: Atypical squamous cells—cannot exclude high grade squamous intraepithelial lesion; AGC-NOS: Atypical glandular cells-not otherwise specified; AGC-FN: atypical glandular cells-favor neoplasia; AIS: adenocarcinoma in situ; HPV: human papillomavirus; LVSI: lymphovascular space involvement; VAIN: vaginal intraepithelial neoplasia; CIN: cervical intraepithelial neoplasia.

**Table 2:** Univariate analysis comparing women with HPV-positive vs. HPV-negative testing.

Independent Variables	HPV-Positive (124) n (%)	HPV-Negative (24) n (%)	*p* Value
Age (median and interquartile ranges, years)	39.5 (34.0–48.0)	47 (35.5–56.5)	0.032
Menopause	18 (14.5)	8 (33.3)	0.027
Nulligravid	59 (47.6)	7 (29.2)	0.098
Smoking habit	29 (23.4)	9 (37.5)	0.148
Vaccinated	10 (8.1)	0 (0.0)	0.151
Conization Type			0.425
CKC	8 (6.5)	3 (12.5)	
Laser C	32 (25.8)	4 (16.7)	
LEEP	84 (67.7)	17 (70.8)	
Cone length (median and interquartile ranges, mm)	16.5 (13.0–20.5)	16.5 (15.0–25.0)	0.485
Pap Test			0.336
Negative	3 (2.4)	2 (8.3)	
ASCUS/LSIL	15 (12.1)	3 (12.5)	
ASCH+	106 (85.5)	19 (79.2)	
Definitive Treatment			0.089
Fertility-sparing	75 (60.5)	10 (41.7)	
Standard treatment	49 (39.5)	14 (58.3)	
Adnexa			0.094
Salpingo-oophorectomy	30 (24.2)	11 (45.8)	
Salpingectomy	13 (10.5)	2 (8.3)	
Positive LVS	9 (7.3)	7 (29.2)	0.001
Stage			0.642
1A1	18 (14.5)	5 (20.8)	
1A2	17 (13.7)	4 (16.7)	
AIS	89 (71.8)	15 (62.5)	
Histopatology			0.049
Usual type	104 (83.9)	16 (66.7)	
Other histology type	20 (16.1)	8 (33.3)	
Positive hr-HPV in follow-up	28 (22.6)	1 (4.2)	0.038
Positive Pap test in follow-up			0.415
ASCUS/LSIL	5 (4.0)	0 (0.0)	
ASCH+	5 (4.0)	2 (8.3)	
Negative	114 (91.9)	22 (91.7)	
Recurrence			0.457
In situ disease	5 (4.0)	2 (8.3)	
Invasive disease	4 (3.2)	0 (0.0)	
No recurrence	115 (92.8)	22 (91.7)	
Time to recurrence (mean ± standard deviation)—Months	36.0 ± 23.0	19.5 ± 23.33	0.384

CKC: cold knife conization; LEEP: loop electrosurgical procedure; ASCUS: atypical squamous cells of undetermined significance; LSIL: low-grade squamous intraepithelial lesion; ASCH: atypical squamous cells—cannot exclude high grade squamous intraepithelial lesion; AIS: adenocarcinoma in situ; HPV: human papillomavirus; LVSI: lymphovascular space involvement.

**Table 3:** Cox proportional hazards regression analysis.

Predictor Variables	Coefficient b	Standard Error	Exp(b)	95% CI of Exp(b)
Age	−0.084	0.051	0.91	0.83 to 1.01
Histopatology = other histology	0.66	0.64	1.94	0.54 to 6.94
Menopause	-	-	-	-
Lymphovascular space involvement = yes	0.49	1.00	1.64	0.22 to 11.75
HPV test in follow-up = positive	2.98	0.97	19.74	2.91 to 133.81
Definitive treatment = standard treatment	0.77	0.74	2.17	0.50 to 9.34
HPV status = negative HPV	1.71	1.18	5.56	0.54 to 57.01

**Table 4:** Recurrence types according to HPV genotypes.

Recurrence Type	HPV Genotypes n (%)				
	HPV-16	HPV-18	HPV-45	Other HPVs	Multiple Infections
No recurrence	19 (28.4)	15 (22.4)	5 (7.5)	8 (11.9)	20 (29.9)
In situ recurrence	1 (20)	3 (60)	1 (20)	0 (0.0)	0 (0.0)
Invasive recurrence	1 (25)	3 (75)	0 (0.0)	0 (0.0)	0 (0.0)

HPV: human papillomavirus.

## Data Availability

Data are available upon reasonable request.
